# Supplementary material for: HIVprotI: an integrated web based platform for prediction and design of HIV proteins inhibitors
Source: J Cheminform. 2018 Mar 9;10:12. doi: 10.1186/s13321-018-0266-y (PMC5845081; doi:10.1186/s13321-018-0266-y)
Supplement: Supplementary file 2 — Additional file 2. Source code of HIVProtI web server. [file 13321_2018_266_MOESM2_ESM.zip › HIVprotI_Source-code/hivprotI/weka-3-6-6/doc/weka/core/converters/AbstractFileSaver.html]

AbstractFileSaver


---


|  |  |  |
| --- | --- | --- |
| **Overview** \| **Package** \| **Class** \| **Tree** \| **Deprecated** \| **Index** \| **Help** \| **Weka's home** | |  |
| **PREV CLASS**   **NEXT CLASS** | **FRAMES**    **NO FRAMES**     **All Classes** |  |
| SUMMARY: NESTED \| FIELD \| CONSTR \| METHOD | DETAIL: FIELD \| CONSTR \| METHOD |  |


---


## weka.core.converters Class AbstractFileSaver

```
java.lang.Object
  weka.core.converters.AbstractSaver
      weka.core.converters.AbstractFileSaver
```

**All Implemented Interfaces:**: java.io.Serializable, CapabilitiesHandler, FileSourcedConverter, Saver, EnvironmentHandler, OptionHandler, RevisionHandler

**Direct Known Subclasses:**: ArffSaver, C45Saver, CSVSaver, LibSVMSaver, SerializedInstancesSaver, SVMLightSaver, XRFFSaver

---

``` public abstract class AbstractFileSaver extends AbstractSaver implements OptionHandler, FileSourcedConverter, EnvironmentHandler ```

Abstract class for Savers that save to a file
Valid options are:
-i input arff file   
The input filw in arff format.

-o the output file   
The output file. The prefix of the output file is sufficient. If no output file is given, Saver tries to use standard out.

**Version:**
:   $Revision: 7706 $

**Author:**
:   Richard Kirkby (rkirkby@cs.waikato.ac.nz), Stefan Mutter (mutter@cs.waikato.ac.nz)

**See Also:**: Serialized Form

---

**Field Summary**

**Fields inherited from interface weka.core.converters.Saver**

`BATCH, INCREMENTAL, NONE`


**Constructor Summary**

`AbstractFileSaver()`


| **Method Summary** | |
| --- | --- |
| `void` | `cancel()`             Cancels the incremental saving process. |
| `java.lang.String` | `filePrefix()`             Gets the file name prefix |
| `abstract  java.lang.String` | `getFileDescription()`             to be pverridden |
| `java.lang.String` | `getFileExtension()`             Gets ihe file extension. |
| `java.lang.String[]` | `getFileExtensions()`             Gets all the file extensions used for this type of file |
| `java.lang.String[]` | `getOptions()`             Gets the current settings of the Saver object. |
| `boolean` | `getUseRelativePath()`             Gets whether relative paths are to be used |
| `java.io.BufferedWriter` | `getWriter()`             Gets the writer |
| `java.util.Enumeration` | `listOptions()`             Returns an enumeration describing the available options. |
| `void` | `resetOptions()`             resets the options |
| `void` | `resetWriter()`             Sets the writer to null. |
| `java.lang.String` | `retrieveDir()`             Gets the directory |
| `java.io.File` | `retrieveFile()`             Gets the destination file. |
| `static void` | `runFileSaver(AbstractFileSaver saver, java.lang.String[] options)`             runs the given saver with the specified options |
| `void` | `setDestination(java.io.File file)`             Sets the destination file (and directories if necessary). |
| `void` | `setDestination(java.io.OutputStream output)`             Sets the destination output stream. |
| `void` | `setDir(java.lang.String dir)`             Sets the directory where the instances should be stored |
| `void` | `setDirAndPrefix(java.lang.String relationName, java.lang.String add)`             Sets the directory and the file prefix. |
| `void` | `setEnvironment(Environment env)`             Set the environment variables to use. |
| `void` | `setFile(java.io.File outputFile)`             Sets the destination file. |
| `void` | `setFilePrefix(java.lang.String prefix)`             Sets the file name prefix |
| `void` | `setOptions(java.lang.String[] options)`             Parses a given list of options. |
| `void` | `setUseRelativePath(boolean rp)`             Set whether to use relative rather than absolute paths |
| `java.lang.String` | `useRelativePathTipText()`             Tip text suitable for displaying int the GUI |

**Methods inherited from class weka.core.converters.AbstractSaver**

`getCapabilities, getInstances, getWriteMode, resetStructure, setInstances, setRetrieval, setStructure, writeBatch, writeIncremental`

**Methods inherited from class java.lang.Object**

`equals, getClass, hashCode, notify, notifyAll, toString, wait, wait, wait`

**Methods inherited from interface weka.core.RevisionHandler**

`getRevision`

**Constructor Detail**

### AbstractFileSaver

```
public AbstractFileSaver()
```


**Method Detail**

### resetOptions

```
public void resetOptions()
```

:   resets the options

    :   **Overrides:**: `resetOptions` in class `AbstractSaver`

---


### getWriter

```
public java.io.BufferedWriter getWriter()
```

:   Gets the writer

    :   **Returns:**: the BufferedWriter

---


### resetWriter

```
public void resetWriter()
```

:   Sets the writer to null.

---


### getFileExtension

```
public java.lang.String getFileExtension()
```

:   Gets ihe file extension.

    :   **Specified by:**: `getFileExtension` in interface `FileSourcedConverter` **Specified by:**: `getFileExtension` in interface `Saver` **Overrides:**: `getFileExtension` in class `AbstractSaver`
    :   **Returns:**: the file extension as a string.

---


### getFileExtensions

```
public java.lang.String[] getFileExtensions()
```

:   Gets all the file extensions used for this type of file

    :   **Specified by:**: `getFileExtensions` in interface `FileSourcedConverter`
    :   **Returns:**: the file extensions

---


### retrieveFile

```
public java.io.File retrieveFile()
```

:   Gets the destination file.

    :   **Specified by:**: `retrieveFile` in interface `FileSourcedConverter`
    :   **Returns:**: the destination file.

---


### setFile

```
public void setFile(java.io.File outputFile)
             throws java.io.IOException
```

:   Sets the destination file.

    :   **Specified by:**: `setFile` in interface `FileSourcedConverter` **Specified by:**: `setFile` in interface `Saver` **Overrides:**: `setFile` in class `AbstractSaver`
    :   **Parameters:**: `outputFile` - the destination file. **Throws:**: `java.io.IOException` - throws an IOException if file cannot be set

---


### setFilePrefix

```
public void setFilePrefix(java.lang.String prefix)
```

:   Sets the file name prefix

    :   **Specified by:**: `setFilePrefix` in interface `Saver` **Overrides:**: `setFilePrefix` in class `AbstractSaver`
    :   **Parameters:**: `prefix` - the file name prefix

---


### filePrefix

```
public java.lang.String filePrefix()
```

:   Gets the file name prefix

    :   **Specified by:**: `filePrefix` in interface `Saver` **Overrides:**: `filePrefix` in class `AbstractSaver`
    :   **Returns:**: the prefix of the filename

---


### setDir

```
public void setDir(java.lang.String dir)
```

:   Sets the directory where the instances should be stored

    :   **Specified by:**: `setDir` in interface `Saver` **Overrides:**: `setDir` in class `AbstractSaver`
    :   **Parameters:**: `dir` - a string containing the directory path and name

---


### retrieveDir

```
public java.lang.String retrieveDir()
```

:   Gets the directory

    :   **Specified by:**: `retrieveDir` in interface `Saver` **Overrides:**: `retrieveDir` in class `AbstractSaver`
    :   **Returns:**: a string with the file name

---


### setEnvironment

```
public void setEnvironment(Environment env)
```

:   Set the environment variables to use.

    :   **Specified by:**: `setEnvironment` in interface `EnvironmentHandler`
    :   **Parameters:**: `env` - the environment variables to use

---


### listOptions

```
public java.util.Enumeration listOptions()
```

:   Returns an enumeration describing the available options.

    :   **Specified by:**: `listOptions` in interface `OptionHandler`
    :   **Returns:**: an enumeration of all the available options.

---


### setOptions

```
public void setOptions(java.lang.String[] options)
                throws java.lang.Exception
```

:   Parses a given list of options. Valid option is:

    -i input arff file   
    The input filw in arff format.

    -o the output file   
    The output file. The prefix of the output file is sufficient. If no output file is given, Saver tries to use standard out.

    :   **Specified by:**: `setOptions` in interface `OptionHandler`
    :   **Parameters:**: `options` - the list of options as an array of strings **Throws:**: `java.lang.Exception` - if an option is not supported

---


### getOptions

```
public java.lang.String[] getOptions()
```

:   Gets the current settings of the Saver object.

    :   **Specified by:**: `getOptions` in interface `OptionHandler`
    :   **Returns:**: an array of strings suitable for passing to setOptions

---


### cancel

```
public void cancel()
```

:   Cancels the incremental saving process.

    :   **Overrides:**: `cancel` in class `AbstractSaver`

---


### setDestination

```
public void setDestination(java.io.File file)
                    throws java.io.IOException
```

:   Sets the destination file (and directories if necessary).

    :   **Specified by:**: `setDestination` in interface `Saver` **Overrides:**: `setDestination` in class `AbstractSaver`
    :   **Parameters:**: `file` - the File **Throws:**: `java.io.IOException` - always

---


### setDestination

```
public void setDestination(java.io.OutputStream output)
                    throws java.io.IOException
```

:   Sets the destination output stream.

    :   **Specified by:**: `setDestination` in interface `Saver` **Overrides:**: `setDestination` in class `AbstractSaver`
    :   **Parameters:**: `output` - the output stream. **Throws:**: `java.io.IOException` - throws an IOException if destination cannot be set

---


### setDirAndPrefix

```
public void setDirAndPrefix(java.lang.String relationName,
                            java.lang.String add)
```

:   Sets the directory and the file prefix.
    This method is used in the KnowledgeFlow GUI

    :   **Specified by:**: `setDirAndPrefix` in interface `Saver` **Overrides:**: `setDirAndPrefix` in class `AbstractSaver`
    :   **Parameters:**: `relationName` - the name of the relation to save: `add` - additional string which should be part of the filename

---


### getFileDescription

```
public abstract java.lang.String getFileDescription()
```

:   to be pverridden

    :   **Specified by:**: `getFileDescription` in interface `FileSourcedConverter`
    :   **Returns:**: the file type description.

---


### useRelativePathTipText

```
public java.lang.String useRelativePathTipText()
```

:   Tip text suitable for displaying int the GUI

    :   **Returns:**: a description of this property as a String

---


### setUseRelativePath

```
public void setUseRelativePath(boolean rp)
```

:   Set whether to use relative rather than absolute paths

    :   **Specified by:**: `setUseRelativePath` in interface `FileSourcedConverter`
    :   **Parameters:**: `rp` - true if relative paths are to be used

---


### getUseRelativePath

```
public boolean getUseRelativePath()
```

:   Gets whether relative paths are to be used

    :   **Specified by:**: `getUseRelativePath` in interface `FileSourcedConverter`
    :   **Returns:**: true if relative paths are to be used

---


### runFileSaver

```
public static void runFileSaver(AbstractFileSaver saver,
                                java.lang.String[] options)
```

:   runs the given saver with the specified options

    :   **Parameters:**: `saver` - the saver to run: `options` - the commandline options


---


|  |  |  |
| --- | --- | --- |
| **Overview** \| **Package** \| **Class** \| **Tree** \| **Deprecated** \| **Index** \| **Help** \| **Weka's home** | |  |
| **PREV CLASS**   **NEXT CLASS** | **FRAMES**    **NO FRAMES**     **All Classes** |  |
| SUMMARY: NESTED \| FIELD \| CONSTR \| METHOD | DETAIL: FIELD \| CONSTR \| METHOD |  |


---
